# Free Vibrations of a Trapezoidal Plate with an Internal Line Hinge

**DOI:** 10.1155/2014/252084

**Published:** 2014-02-26

**Authors:** María Virginia Quintana, Ricardo Oscar Grossi

**Affiliations:** ^1^INIQUI-CONICET, Facultad de Ingeniería, Universidad Nacional de Salta, Av. Bolivia 5150, 4400 Salta, Argentina; ^2^Facultad de Ingeniería e Informática, Universidad Católica de Salta, Pellegrini 790, Salta, Argentina

## Abstract

This paper deals with a general variational formulation for the determination of natural frequencies and mode shapes of free vibrations of laminated thin plates of trapezoidal shape with an internal line hinge restrained against rotation. The analysis was carried out by using the kinematics corresponding to the classical laminated plate theory (CLPT). The eigenvalue problem is obtained by employing a combination of the Ritz method and the Lagrange multipliers method. The domain of the plate is transformed into a rectangular domain in the computational space by using nonorthogonal triangular coordinates and the transverse displacements are approximated with a set of simple polynomials automatically generated and expressed in the triangular coordinates. The developed algorithm allows obtaining approximate analytical solutions for mentioned plate with different geometries, aspect ratio, position of the line hinge, and boundary conditions including translational and rotational elastically restrained edges. It allows studying the influence of the mentioned line on the vibration frequencies and respective mode shapes. The algorithm can easily be programmed and it is numerically stable. Additionally, as a particular case, the results of triangular plates can be easily generated.

## 1. Introduction

Anisotropic plates, particularly those made of fiber-reinforced composite materials, are widely used in numerous industrial and engineering disciplines such as mechanical, aerospace, electronics, optical, and structural fields. The rapid increase in the industrial use of this type of structural elements has brought with it the need to develop analytical and numerical techniques that are appropriate for the analysis of its mechanical behavior. In particular, trapezoidal plates are widely used as structural elements, as well as single elements or as part of more complex structures. In many cases, for these structural components, the rapid and efficient determination of the natural vibration frequencies and their associated mode shapes is essential in the design and performance evaluation. Moreover, the resonant frequencies and mode shapes of these plates are used to set the corresponding dynamic response of more complex systems.

Most published papers which analyze the free vibrations of anisotropic plates with elastically restrained boundaries concern rectangular plates. There is a less number of works related to dynamic analysis of anisotropic plates of trapezoidal forms [1–7].

The presence of an internal line hinge in a plate can be used to facilitate the opening of gates and to represent internal cracks. Rotational springs located in the line hinge can be used for modeling a fracture plane with an arbitrary depth [8, 9].

The first known solution theory based on shear deformation of first order for the vibration of rectangular plates with an internal line hinge has been provided by [10]. The authors used the method of Levy and the state-space technique to solve this problem and obtain the frequency coefficients values. The method is only applicable to rectangular plates with at least two parallel edges simply supported. More recently a discrete method to analyze the free vibration problem of moderately thick rectangular plates with an intermediate line hinge and arbitrary edge conditions has been presented [11]. Quintana and Grossi [12] dealt with the study of free transverse vibrations of isotropic rectangular plates with an internal line hinge and elastically restrained boundaries. The problem was solved employing a combination of the Ritz method and the Lagrange multiplier method. However, in these works, anisotropic materials were not analyzed.

Hamilton's principle has been used for the derivation of equations of motion and its associated boundary and transition conditions of anisotropic plates with an arbitrarily located internal line hinge with elastics supports and piecewise-smooth boundaries elastically restrained against rotation and translation among other complicating effects [13]. In the same manner, the model has been extended to analyze several anisotropic plates with intermediate lines hinge [14]. Values of the coefficients of frequencies and mode shapes were obtained by applying the Ritz method. Nevertheless, the numerical results obtained in the previously quoted papers correspond to rectangular plates.

The study of trapezoidal plates through the Ritz method presents the difficulty of the construction of simple and adequate approximation functions that can be applied to the domain of the plate [15, 16]. When these plates also have an internal line hinge elastically restrained against rotation, the mathematical structure of the transition conditions becomes more complex. These transition conditions give rise to several problems in the rational choice of the coordinate functions. In fact, the most critical feature of the Ritz method is regarding the choice of the mentioned functions. So in this paper, only the essential transition condition along the line hinge is taken into account with the Lagrange multipliers.

According to the statement in the preceding paragraphs, the objective of this paper is to propose a general algorithm to obtain approximate analytical solutions for the study of the free vibrations of trapezoidal plates with an intermediate line hinge elastically restrained against rotation. The procedure is based on the Ritz method in combination with the Lagrange multipliers method and covers two aspects. The first is the approximation of the plate geometry through triangular coordinates and the second aspect is the approximation of the transverse displacement with simple polynomials generated automatically from a basis polynomial.

The obtained analytical solution has a great advantage since it allows studying the influence of the position of the line internal line hinge and the degree of rotational restriction on the vibration frequencies. To demonstrate the validity and efficiency of the developed algorithm, results of a convergence study are included, several numerical examples not previously treated are presented, and some particular cases are compared with results presented by other authors.

## 2. Mathematical Formulation

### 2.1. Geometrical and Mechanical Characteristics of the Plate

**Figure 1:**
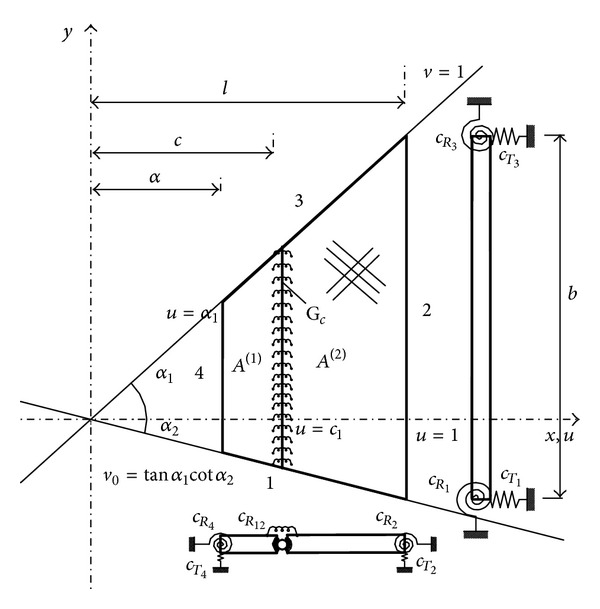
General description of the mechanical system under study.

**Figure 2:**
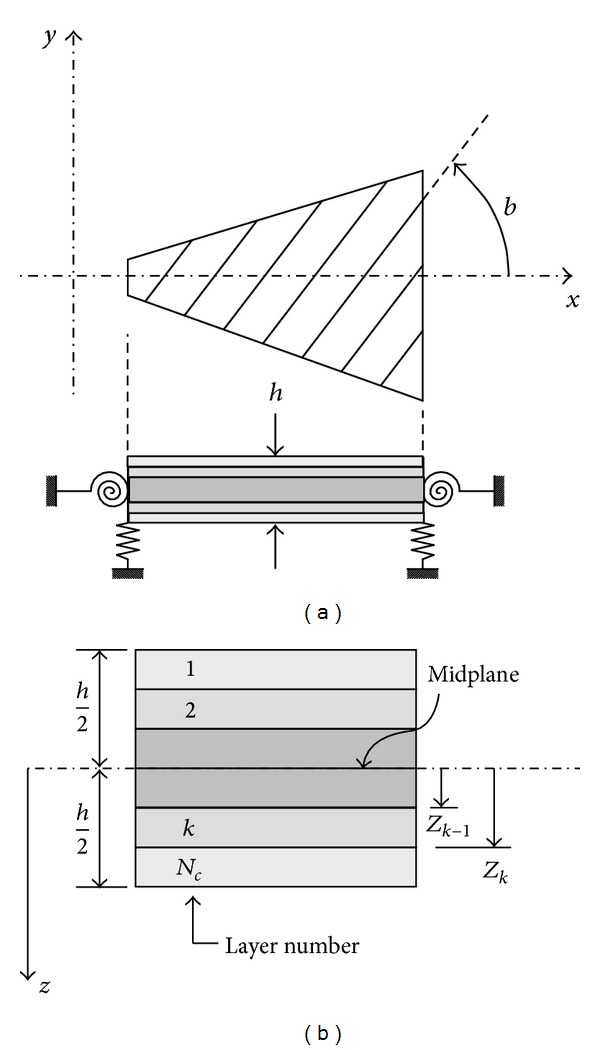
Mechanical system. (a) Elastic restraints and angle of fibers orientation. (b) Profile and laminate stacking sequence.

The general scheme of the analyzed composite trapezoidal plate with an intermediate line hinge is shown in Figure 1. The laminate thickness is *h* and, in general, it consists of layers of unidirectional fibers composite material (Figure 2(b)). The lamination scheme is symmetric with respect to the midplane. The angle of fibers orientation is denoted by *β*, measured from *x*-axis to the fibers direction as shown in Figure 2(a). The rotational and translational restraints are, respectively, characterized by the springs constants *c*_*R*_12__,  *c*_*R*_*i*__ and *c*_*T*_*i*__  (*i* = 1,…, 4).

The present study is based on the kinematics corresponding to the classical laminated plate theory (CLPT). For free plate vibration, it is possible to suppose that the displacement is given by harmonic functions of the time; that is,

(1) $$\begin{array}{c} w\left(x,y,t\right)=W\left(x,y\right)\cos\omega t, \end{array}$$

where *ω* is the radian frequency of the plate, and the maximum kinetic energy of the described plate can be expressed in rectangular coordinates by

(2) $$\begin{array}{c} T_{\max}=\frac{\rho h\omega^{2}}{2}\overset{2}{\underset{p=1}{\sum }}\iint_{A^{\left(p\right)}}{\left(W^{\left(p\right)}\left(x,y\right)\right)}^{2}dx\;dy, \end{array}$$

where *ρ* denotes the mass density of the material of the plate and *W* ^(*p*)^(*x*, *y*),  *p* = 1,2 the amplitudes of the transverse displacements which respectively correspond to the subdomains *A* ^(1)^ and *A* ^(2)^.

Taking into account the assumptions of the CLPT, the maximum strain energy of the mechanical system is given by

(3) $$\begin{array}{c} U_{\max}=U_{P,\max}+U_{R,\max}+U_{T,\max}, \end{array}$$

where *U*_*P*,max⁡_ is the maximum strain energy due to plate bending, which in Cartesian coordinates is given by

(4) UP,max⁡=12∑p=12∬A(p)[D11(p)(∂2W(p)∂x2)2+2D12(p)∂2W(p)∂x2∂2W(p)∂y2+D22(p)(∂2W(p)∂y2)2 +4(D16(p)∂2W(p)∂x2+D26(p)∂2W(p)∂y2)∂2W(p)∂x∂y +4D66(p)(∂2W(p)∂x∂y)2]dx dy,

where the coefficients *D*_*ij*_^(*p*)^, *p* = 1, 2,  *i*, *j* = 1,2, 6 are the bending, twisting, and bending-twisting coupling rigidities, which are given by

(5) $$\begin{array}{c} D_{ij}^{\left(p\right)}=\frac{1}{3}\overset{N_{c}}{\underset{k=1}{\sum }}{\left({\overset{-}{Q}}_{ij}^{\left(k\right)}\right)}^{\left(p\right)}\left(z_{k+1}^{3}-z_{k}^{3}\right), \end{array}$$

where *z*_*k*+1_, *z*_*k*_ are the distances from the middle plate to the top and bottom of the *k*th layer (see Figure 2(b)), *N*_*c*_ is the total number of layers in the laminate, and ${\left({\overset{-}{Q}}_{ij}^{\left(k\right)}\right)}^{\left(p\right)}$ are the reduced transformed rigidities for a plane state of tensions (see, e.g., [18]).

The maximum strain energy *U*_*T*,max⁡_ stored in translational springs of constants *c*_*T*_(*s*) at the plate edges is given by

(6) $$\begin{array}{c} U_{T,\max}=\frac{1}{2}\overset{4}{\underset{i=1}{\sum }}\int_{\partial A}c_{T_{i}}^{}{\left(W\left(s\right)\right)}^{2}ds. \end{array}$$

On the other hand, the maximum strain energy *U*_*R*,max⁡_ stored in rotational springs of constants *c*_*R*_(*s*) and *c*_*R*_12__(*s*) at the plate edges and at the internal line hinge is, respectively, given by

(7) UR,max⁡=12∑i=14∫∂AcRi(∂W∂xnxi+∂W∂ynyi)2ds +12∫TccR12(∂W(1)∂x−∂W(2)∂x)ds,

where *n*_*x*_*i*__ and *n*_*y*_*i*__ denote the components of the outward normal *n*_*i*_ of ∂*A*_*i*_ which are given by

(8) $$\begin{array}{c} n_{x_{1}}^{}=\sin\alpha_{2}\;\;n_{y_{1}}^{}=-\cos\alpha_{2},\\ n_{x_{2}}^{}=1\;\;n_{y_{2}}^{}=0,\\ n_{x_{3}}^{}=-\sin\alpha_{1}\;\;n_{y_{3}}^{}=\cos\alpha_{1},\\ n_{x_{4}}^{}=-1\;\;n_{y_{4}}^{}=0. \end{array}$$

### 2.2. Triangular Nonorthogonal Coordinates

The actual plate of trapezoidal plan-form is mapped onto a rectangular one, using a coordinate transformation between the rectangular Cartesian and triangular nonorthogonal coordinates, according to the following expressions [15, 16]:

(9) $$\begin{array}{c} u=\frac{x}{l},\;\;v=\frac{y}{x\;\text{cot}\;\alpha_{1}}, \end{array}$$

where tan*α*_1_ is the slope of the upper side of the plate (see Figure 1). The relationships between the partial derivatives in both coordinates systems are given by

(10) [∂(·)∂x∂(·)∂y]=J−1[∂(·)∂u∂(·)∂v]=[J22|J|−J12|J|−J21|J|J11|J|][∂(·)∂u∂(·)∂v],

where **J**  is the Jacobian matrix of the geometrical mapping given by

(11) $$\begin{array}{c} J=\left[\begin{array}{cc} J_{11} & J_{12}\\ J_{21} & J_{22} \end{array}\right]=\left[\begin{array}{cc} \frac{\partial x}{\partial u} & \frac{\partial y}{\partial u}\\ \frac{\partial x}{\partial v} & \frac{\partial y}{\partial v} \end{array}\right]=\left[\begin{array}{cc} l & vl\tan\alpha_{1}\\ 0 & ul\tan\alpha_{1} \end{array}\right] \end{array}$$

and |**J**| denoted the determinant of the matrix (11). In the same manner, the relationship between the second partial derivatives are obtained.

The maximum kinetic and strain energies of the mechanical system can now be expressed in the non-orthogonal triangular coordinates and are, respectively, given by

(12) Tmax⁡=ρhl2ω2tanα12∑p=12∬A(p)u(W(p))2du dv,UP,max⁡=D0tanα12l2 ×∑p=12∫v01∫up−1up[S1(p)u(∂2W(p)∂u2)2        +S2(p)u3(∂2W(p)∂v2)2        +2S3(p)u∂2W(p)∂u2∂2W(p)∂v2        +4S4(p)∂2W(p)∂u2∂2W(p)∂u∂v        +4S5(p)u2∂2W(p)∂v2∂2W(p)∂u∂v        +4S6(p)u(∂2W(p)∂u∂v)2        +4S7(p)u∂2W(p)∂u2∂W(p)∂v        +4S8(p)u3∂2W(p)∂v2∂W(p)∂v        +8S9(p)u2∂2W(p)∂u∂v∂W(p)∂v        +4S10(p)u3(∂W(p)∂v)2]du dv,

where *W* = *W*(*u*, *v*) = *W*(*x*, *y*)/*l*, *du* *dv* = *dx* *dy*/(*ul* ^2^tan*α*_1_), *u*_0_ = *a*_*l*_, *u*_1_ = *c*_*l*_, *u*_2_ = 1, and *S*_*i*_  (*i* = 1,…, 10) are functions which depend on the parameters of the problem which correspond to the geometry and the material properties. The mentioned coefficients are defined in Appendix A.

The maximum strain energies stored in the translational and rotational springs at the plate edges become

(13) UT,max⁡=D0tanα12l2∑p=12(T1cos⁡α2tanα1∫up−1up(W(p))2|v=v0du       +T3sinα1∫up−1up(W(p))2|v=1du) +T2∫v01(W(2))2|u=1dv+alT4∫v01(W(1))2|u=aldv,

(14) UR,max⁡ =D0tanα12l2  ×{∑p=12[R1∫up−1up(d11(∂W(p)∂u)2          +d12u2(∂W(p)∂v)2          +2d13u          ×∂W(p)∂u∂W(p)∂v)|v=v0du     +R3∫up−1up(d31(∂W(p)∂u)2          +d32u2(∂W(p)∂v)2          +2d33u∂W(p)∂u          ×∂W(p)∂v)|v=1du]  +R2∫v01(d21(∂W(2)∂u)2+d22u2(∂W(2)∂v)2      +2d23u∂W(2)∂u∂W(2)∂v)|u=1dv  +alR4∫v01(d21(∂W(1)∂u)2+d22u2(∂W(1)∂v)2       +2d23u∂W(1)∂u∂W(1)∂v)|u=aldv  +clR12∫v01[∑p=12(d21(∂W(p)∂u)2+d22u2(∂W(p)∂v)2         +2d23u∂W(p)∂u∂W(p)∂v)|u=cl       −2d21∂W(1)∂u∂W(2)∂u       +d22u2∂W(1)∂v∂W(2)∂v       −d23u(∂W(1)∂u∂W(2)∂v          +∂W(1)∂v∂W(2)∂u)]|u=cldv},

where *R*_*i*_ = *c*_*R*_*i*__ *l*/*D*_0_, *R*_12_ = *c*_*R*_12__ *l*/*D*_0_, and *T*_*i*_ = *c*_*T*_*i*__ *l* ^3^/*D*_0_,  *i* = 1,…, 4,

(15) $$\begin{array}{c} d_{11}=\frac{\text{si}{\text{n}}^{2}\alpha_{2}}{\cos\alpha_{2}\tan\alpha_{1}},\\ d_{12}=\frac{1}{\tan\alpha_{1}}\left(\frac{\cos\alpha_{2}}{\text{ta}{\text{n}}^{3}\alpha_{1}}+2\frac{v_{0}\sin\alpha_{2}}{\text{ta}{\text{n}}^{2}\alpha_{1}}+\frac{v_{0}^{2}\text{si}{\text{n}}^{2}\alpha_{2}}{\cos\alpha_{2}\tan\alpha_{1}}\right),\\ d_{13}=-\left(\frac{\sin\alpha_{2}}{\text{ta}{\text{n}}^{2}\alpha_{1}}+\frac{v_{0}\text{si}{\text{n}}^{2}\alpha_{2}}{\cos\alpha_{2}\tan\alpha_{1}}\right),\\ d_{21}=1,\;\;d_{22}=v^{2},\;\;d_{23}=-v,\;\;d_{31}=\sin\alpha_{1},\\ d_{32}=\frac{\cos\alpha_{1}}{\text{ta}{\text{n}}^{3}\alpha_{1}}+2\frac{\cos\alpha_{1}}{\tan\alpha_{1}}+\sin\alpha_{1},\\ d_{33}=-\left(\frac{\cos\alpha_{1}}{\tan\alpha_{1}}+\sin\alpha_{1}\right). \end{array}$$

## 3. The Ritz and Lagrange Multipliers Methods (*R* & LMM)

When a variational formulation is used to study the behaviour of a structure obtained by joining several components together, several transition conditions arises a correspondence of the presence of the junctions of the structural components. When employing the Ritz method, fortunately it is not necessary to subject the coordinate functions to the natural boundary conditions [18, 19]. This concept can be extended to the transition conditions and is particularly true in the case of a rectangular plate with an internal line hinge [12].

According to [12, 13], the only essential transition condition of the problem under study is that ensures the continuity of transverse displacement along the line hinge and which imposes the analytical condition

(16) $$\begin{array}{c} W^{\left(1\right)}\left(c_{l},v\right)-W^{\left(2\right)}\left(c_{l},v\right)=0,\;\forall v\in \left(v_{0},1\right). \end{array}$$

It is difficult to construct a simple and adequate deflection function which can be applied to the entire domain of the plate and to show the continuity of displacement and the discontinuities of the slope crossing the line hinge. One way to eliminate the requirement given by (16) on the coordinate functions is to perform the process of minimization over a increased energy functional using subsidiary conditions. The transition conditions (16) can be incorporated in the energy functional by means of a suitable Lagrange multiplier [20, 21]. This leads to the following functional:

(17) $$\begin{array}{c} L\left(W,\lambda \right)=\Pi \left(W\right)+\langle G\left(W\right),\lambda \rangle , \end{array}$$

where

(18) $$\begin{array}{c} G\left(W\right)\equiv W^{\left(1\right)}-W^{\left(2\right)}=0,\;\forall \left(x,y\right)\in \Gamma_{c},\\ \langle G\left(W\right),\lambda \rangle =\overset{}{\underset{\Gamma_{c}}{\int }}\lambda \left(s\right)\left(W^{\left(1\right)}\left(s\right)-W^{\left(2\right)}\left(s\right)\right)ds,\\ \Pi =U_{\max}^{}-T_{\max}, \end{array}$$

and *λ* is the Lagrange multiplier. It must be noted that in this case, the Lagrange multiplier is a function.

Now, the idea is to minimize the functional (17) over the deflection functions which satisfy only the geometrical boundary conditions on the subdomains *A* ^(1)^ and *A* ^(2)^.

### 3.1. Approximating Functions

The approximating functions are chosen assuming that we have two independent subdomains and that these functions verify the corresponding essential boundary conditions. In the present paper, the transverse deflections for *A* ^(1)^ and *A* ^(2)^ are represented by means of products of one-dimensional polynomials in each of the triangular coordinates, as follows:

(19) $$\begin{array}{c} W^{\left(p\right)}\left(u,v\right)=\overset{M}{\underset{i=1}{\sum }}\overset{N}{\underset{j=1}{\sum }}c_{ij}^{\left(p\right)}p_{i}^{\left(p\right)}\left(u\right)q_{j}^{\left(p\right)}\left(v\right),\;p=1,2, \end{array}$$

where *c*_*ij*_^(·)^ are the unknown coefficients to be determined by the Ritz method. The sets of polynomials {*p*_*i*_^(1)^(*u*)},  {*q*_*j*_^(1)^(*v*)},  {*p*_*i*_^(2)^(*u*)}, and {*q*_*j*_^(2)^(*v*)} are generated recursively by starting with polynomials which satisfy the essential boundary conditions of the equivalent beam in each triangular coordinate. These polynomials depend on the boundary conditions and when the edges are free or have rotational or translational restraints, all the boundary conditions are natural so it is possible to ignore the boundary conditions in the construction of the first polynomial. Similarly, it is possible to ignore the restriction (16) on the interface and to consider it as a free edge.

The higher members of the described sets are automatically generated using the following procedure, for example:

(20) $$\begin{array}{c} p{\left(u\right)}_{i}^{\left(p\right)}=p{\left(u\right)}_{1}^{\left(p\right)}u^{i-1},\;i=2,\ldots ,M. \end{array}$$

The polynomials set along the *v* direction are generated using the same procedure.

### 3.2. Lagrange Multiplier Function

Since the Lagrange multiplier is a function, it can be approximated by using the following polynomial expression:

(21) $$\begin{array}{c} \lambda \left(v\right)=\overset{N}{\underset{i=1}{\sum }}c_{i}^{\left(\lambda \right)}v^{i-1}, \end{array}$$

where *c*_*i*_^(*λ*)^are the unknown coefficients.

### 3.3. Eigenvalue Problem

Application of the Ritz method in combination with the Lagrange multiplier method requires the minimization of functional (17). In consequence, we have [20]

(22) $$\begin{array}{c} \delta L\left(W,\lambda \right)=0. \end{array}$$

Equation (22) can be recovered by setting the partial variations to zero [20]. This leads to the following:

(23) $$\begin{array}{c} \delta L_{W}\left(W,\lambda ;U\right)=0⟹\delta \Pi \left(W\right)+\lambda \delta G\left(W\right)=0,\\ \delta L_{\lambda }\left(W,\lambda ;\eta \right)=0⟹G\left(W\right)=0. \end{array}$$

Substituting (19) and (21), into (23) an eigenvalue problem is obtained, which is

(24) $$\begin{array}{c} \left(\left[K\right]-\Omega^{2}\left[M\right]\right)\left\{c\right\}=\left\{0\right\}, \end{array}$$

where $\Omega =\omega l^{2}\sqrt{\rho h/D_{0}}$ is the dimensionless frequency parameter and the matrix [*K*] and [*M*] are given by

(25) $$\begin{array}{c} \left[K\right]=\left[\begin{array}{ccc} \left[K^{\left(1\right)}\right] & \left[K^{\left(12\right)}\right] & \left[K^{\left(1\lambda \right)}\right]\\ & \left[K^{\left(2\right)}\right] & \left[K^{\left(2\lambda \right)}\right]\\ \text{sim}. & & \left[0\right] \end{array}\right],\\ \left[M\right]=\left[\begin{array}{ccc} \left[M^{\left(1\right)}\right] & \left[0\right] & \left[0\right]\\ & \left[M^{\left(2\right)}\right] & \left[0\right]\\ \text{sim}. & & \left[0\right] \end{array}\right]. \end{array}$$

The expressions of the elements of these matrixes are given in Appendix B.

Equation (24) yields an algebraic equation whose zeros give the natural frequencies of the mechanical system under study. Back substitution yields the coefficient vectors {**c**}, and finally substitution of these coefficient vectors into (19) gives the corresponding mode shapes of the plate.

## 4. Results and Discussion

In order to establish the accuracy and applicability of the approach developed and discussed in the previous sections, numerical results were computed for a number of plate problems for which comparison values were available in the literature. Additionally, new numerical results were generated for trapezoidal plates with an internal line hinge and different boundary conditions.

The terminology to be used throughout the remainder of the paper for describing the boundary conditions of the plate considered will now be introduced. The designation CSFS, for example, identifies a plate with the edges 1 clamped, 2 simply supported, 3 free, and 4 simply supported (see Figure 1). For triangular plates, edge 4 disappears. When the plate's edges have rotational and/or translational restraints, the restraints parameters are specifically indicated in each case.

Table 1 shows the first five values of the frequency parameters $\Omega =\omega /(2\pi )l^{2}\sqrt{\rho h/D_{0}}$,  *D*_0_ = *E*_1_^(1)^ *h* ^3^/12(1 − *ν*_12_^(1)^ *ν*_21_^(1)^) for anisotropic trapezoidal plate with a free internal line hinge located at different positions. The plates have different geometrical properties and are subjected to different boundary conditions. The anisotropic material is characterized by the constants ${\overset{-}{D}}_{11}^{\left(p\right)}=1.0$,  ${\overset{-}{D}}_{12}^{\left(p\right)}=0.100812496$,  ${\overset{-}{D}}_{16}^{\left(p\right)}=-0.2433353$,  ${\overset{-}{D}}_{26}^{\left(p\right)}=-0.0120837$, and ${\overset{-}{D}}_{66}^{\left(p\right)}=0.0948810$.

Table 2 depicts the variation of the frequency parameters $\Omega^{**}=\omega /(2\pi )l^{2}\sqrt{\rho h/H}$ with *D*_0_ = *H* = *ν*_12_^(1)^ *D*_22_^(1)^ + 2*D*_66_^(1)^ for FCFF trapezoidal plates (tan*α*_1_ = 1/3, tan*α*_2_ = 0) with an internal line hinge elastically restrained against rotation located at different positions. The plate consists in a single boron-epoxy layer with *β* = 0. The physical properties of the layer are given by ${\overset{-}{D}}_{11}^{\left(p\right)}=15.637$,  ${\overset{-}{D}}_{22}^{\left(p\right)}=0.91160$, and ${\overset{-}{D}}_{66}^{\left(p\right)}=0.35642$. In this table, it can be observed the effect of the rotational restriction *R*_12_ over the vibration frequencies. Additionally, the frequency parameters of the triangular plate are compared with results of [17] when the intermediate elastic restriction *R*_12_ → *∞* is adopted. It can be observed that the present solutions are in good agreement, from an engineering viewpoint.

**Figure 3:**
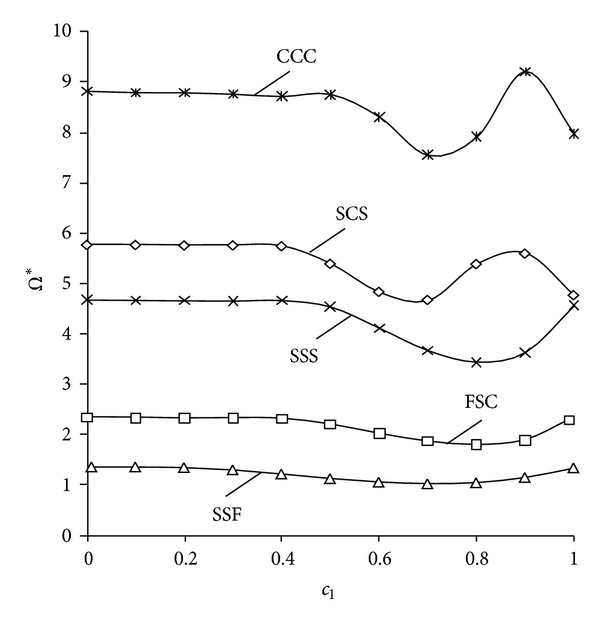
Variation of the fundamental frequency parameters $\Omega^{*}=\omega /(2\pi )l^{2}\sqrt{\rho h/D_{0}}$ with respect to the position of the line hinge *c*_*l*_ for isosceles triangular isotropic plates with different boundary conditions (tan*α*_1_ = −tan*α*_2_ = 0.5, *R*_12_ = 0).

On the other hand, effect of the position of the line hinge is presented in Figure 3. This figure shows the variation of the fundamental frequency parameters $\Omega^{*}=\omega /(2\pi )l^{2}\sqrt{\rho h/D}$ with *D*_0_ = *D* = *Eh* ^3^/[12(1 − *ν* ^2^)] of an isosceles triangular isotropic plate with respect to parameter *c*_*l*_. Plates with several types of combinations of edge conditions are taken into account. It can be observed, in all cases, that the effects due to the presence of the line hinge over the frequency increase when the position of the line hinge is away from the left side of the plate; that is, *c*_*l*_ > 0.4.

**Figure 4:**
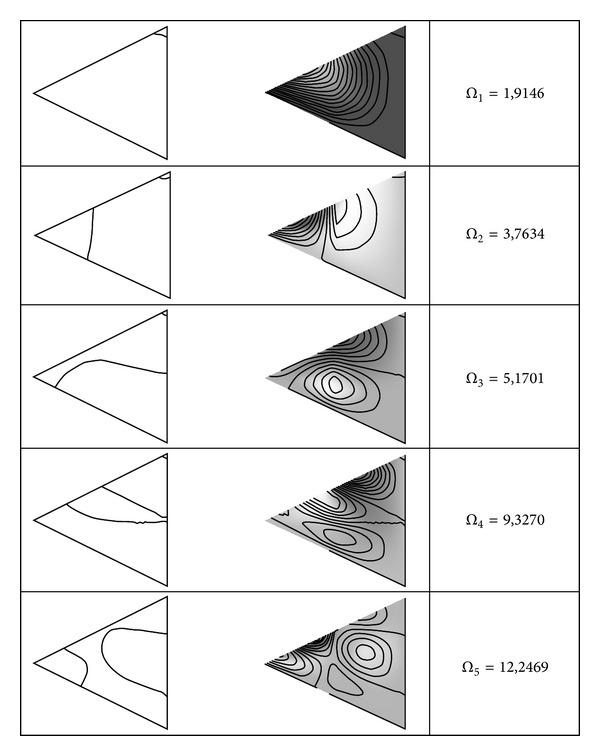
First five values of the frequency parameter $\Omega =\omega /(2\pi )l^{2}\sqrt{\rho h/D_{0}}$ nodal patterns and mode shapes for a SCF anisotropic triangular plate (tan*α*_1_ = −tan*α*_2_ = 0.5) with a line hinge (*R*_12_ = 0) located at *c*_*l*_ = 0.5.

Finally, some representative mode shapes and nodal patterns for a triangular SCF triangular anisotropic plate (tan*α*_1_ = −tan*α*_2_ = 0.5) with a free internal line hinge located in *c*_*l*_ = 0.5 are presented in Figure 4. It can be observed that the line hinge presence introduces significant deformations along this line. The physical properties are the same that have been used to generate Table 2.

## 5. Conclusion

This paper presents a simple, accurate, and general algorithm for the determination of frequencies and modal shapes of natural vibrations of trapezoidal and triangular symmetrically laminated plates with an arbitrarily located internal line hinge. The approach is based on a combination of the Ritz method and the Lagrange multipliers method using the kinematic corresponding to de CLPT plate theory and non-orthogonal right triangular coordinates to express the geometry of the plate in a simple form. The transverse deflection is approximated by means of simple polynomials. The algorithm allows a unified treatment of symmetrically laminated plates with several trapezoidal or triangular planform, different boundary conditions, including edges elastically restrained against rotation and translation.

Sets of numerical results are given in tabular and graphical forms illustrating the influence of the position of the internal line hinge and the degree of the corresponding rotational restriction on the vibration frequencies and respective mode shapes.

Finally, it is important to note that the proposed method can be easily extended for application to static and stability analysis. It can also be generalized to study trapezoidal plates with non-symmetrical stacking sequence about the midplane.

## Figures and Tables

**Table 1:** The first five values of the frequency parameter $\Omega =\omega /(2\pi )l^{2}\sqrt{\rho h/D_{0}}$ for anisotropic trapezoidal plates with an internal line hinge located at different positions and subject to different boundary conditions.

tan*α*_1_ = tan*α*_2_	*a*_*l*_	*c*_*l*_	Ω_1_	Ω_2_	Ω_3_	Ω_4_	Ω_5_
			SCSS				
0,5	0	0,5	4,0444	9,8133	11,9368	16,1093	16,8048
0,4	0,2	0,6	3,0037	7,4804	9,8142	13,1061	13,9349
0,3	0,4	0,7	2,3947	5,6939	8,4165	10,7334	11,8879

			SCCS				
0,5	0	0,5	5,7756	11,6366	12,8590	18,0203	19,1298
0,4	0,2	0,6	4,0323	9,1264	10,6307	14,0167	15,1435
0,3	0,4	0,7	2,9223	6,8977	8,6742	11,9163	12,9837

			SSCF				
0,5	0	0,5	4,8013	9,3880	10,0453	15,4360	17,0102
0,4	0,2	0,6	3,3904	7,7580	8,4436	12,0781	12,7893
0,3	0,4	0,7	2,3467	4,8566	6,1935	8,3330	10,2203

			SCFF				
0,5	0	0,5	1,9146	3,7634	5,1701	9,3270	12,2469
0,4	0,2	0,6	1,2156	2,3263	4,0540	7,1127	8,3748
0,3	0,4	0,7	0,7525	1,9975	3,1531	4,5058	6,6353

**Table 2:** The first five values of the frequency parameter $\Omega^{**}=\omega /(2\pi )l^{2}\sqrt{\rho h/H}$ for boron-epoxy (*β* = 0) FCFF trapezoidal plates with an internal line hinge located at different positions (tan*α*_1_ = 1/3, tan*α*_2_ = 0).

*a*_*l*_	*R*_12_	Ω_1_**	Ω_2_**	Ω_3_**	Ω_4_**	Ω_5_**
		*c*_*l*_ = 0.5				
0	∞	3,7681	14,2391	20,7233	36,8583	50,4389
	Reference [17]	3,7682	14,2395	20,7250	36,8596	50,4602
	1000	3,7592	14,1806	20,5222	36,7239	50,3837
	100	3,6818	13,6411	19,2421	35,7874	49,9690
	10	3,0694	10,3632	17,1129	33,2800	48,3122
	5	2,6281	9,2487	16,8830	32,7645	47,8666
	1	1,4592	7,9784	16,6962	32,2439	47,3974

		*c*_*l*_ = 0.6				
0.2	∞	4,5238	16,0582	24,8369	42,1620	64,6896
	1000	4,5071	16,0437	24,4224	41,7750	64,6747
	100	4,3630	15,9091	21,7174	39,2726	64,5875
	10	3,3755	13,7284	16,9941	34,2600	64,4185
	5	2,7871	12,5570	16,7404	33,4563	64,3882
	1	1,4681	11,2472	16,5925	32,6963	64,3580

		*c*_*l*_ = 0.7				
0.4	∞	7,0576	18,5364	40,1040	53,4753	81,6813
	1000	7,0209	18,5194	39,1366	52,7708	81,2581
	100	6,7104	18,4066	33,2704	48,4565	79,1987
	10	4,8364	18,0067	22,2985	40,7709	76,8733
	5	3,8790	17,7903	20,6121	39,6158	76,6110
	1	1,9656	17,2753	19,3033	38,5349	76,3799

## Appendices

### A.

Consider

(A.1) S1(i)=D−11(i)u,S2(i)=D−11(i)v4+D−22(i)cot4 α1+(D−12(i)+2D−66(i))2v2cot 2α1−4D−16(i)v3cot α1−4D−26(i)v cot3 α1,S3(i)=D−11(i)v2+D−12(i)cot2 α1−2D−16(i)v cot α1,S4(i)=−D−11(i)v+D−16(i) cot α1,S5(i)=−D−11(i)v3−D−12(i)v cot2 α1−2D−66(i)v cot2 α1+3D−16(i)v2cot α1+D−26(i)cot3α1,S6(i)=D−11(i)v2+D−66(i)cot2α1−2D−16(i)v cot α1,S7(i)=−S4(i),S8(i)=−S5(i),S9(i)=−S6(i),S10(i)=S6(i),D−ij(p)=Dij(p)D0, p=1,2,D0=E1(1)h312(1−ν12(1)ν21(1)).

### B.

Consider

(B.1) Kijmn(p)=∫up−1up∫v01[S1(p)uPik(p,p)(2,2)Qjl(p,p)(0,0)     +S2(p)u3Pik(p,p)(0,0)Qjl(p,p)(2,2)     +S3(p)u(Pik(p,p)(0,2)Qjl(p,p)(2,0)        +Pik(p,p)(2,0)Qjl(p,p)(0,2))     +2S4(p)u(Pik(p,p)(1,2)Qjl(p,p)(1,0)         +Pik(p,p)(2,1)Qjl(p,p)(0,1))     +2S5(p)u2(Pik(p,p)(1,0)Qjl(p,p)(1,2)         +Pik(p,p)(0,1)Qjl(p,p)(2,1))     +4S6(p)uPik(p,p)(1,1)Qjl(p,p)(1,1)     +2S7(p)u(Pik(p,p)(0,2)Qjl(p,p)(1,0)         +Pik(p,p)(2,0)Qjl(p,p)(0,1))     +2S8(p)u3(Pik(p,p)(0,0)Qjl(p,p)(1,2)         +Pik(p,p)(0,0)Qjl(p,p)(2,1))     +4S9(p)u2(Pik(p,p)(0,1)Qjl(p,p)(1,1)         +Pik(p,p)(1,0)Qjl(p,p)(1,1))     +4S10(p)u3Pik(p,p)(0,0)Qjl(p,p)(1,1)]du dv +T1cos⁡α2tanα1 ×∫up−1upPik(p,p)(0,0)Qjl(p,p)(0,0)|v=v0du +u2(p−1)T2(3−p)/p ×∫v01Pik(p,p)(0,0)Qjl(p,p)(0,0)|u=u2(p−1)dv  +T3sinα1∫up−1uppPik(p,p)(0,0)Qjl(p,p)(0,0)|v=1du  +R1∫up−1up[d11Pik(p,p)(1,1)Qjl(p,p)(0,0)       +d12u2Pik(p,p)(0,0)Qjl(p,p)(1,1)       +d13u(Pik(p,p)(1,0)Qjl(p,p)(0,1)          +Pik(p,p)(0,1)          ×Qjl(p,p)(1,0))]|v=v0dv +R3∫up−1up[d31Pik(p,p)(1,1)Qjl(p,p)(0,0)      +d32u2Pik(p,p)(0,0)Qjl(p,p)(1,1)      +d33u(Pik(p,p)(1,0)Qjl(p,p)(0,1)         +Pik(p,p)(0,1)Qjl(p,p)(1,0))]|v=1du +u2(p−1)R2(3−p)/p∫v01[d21Pik(p,p)(1,1)Qjl(p,p)(0,0)          +d22u2Pik(p,p)(0,0)Qjl(p,p)(1,1)          +d23u(Pik(p,p)(1,0)Qjl(p,p)(0,1)             +Pik(p,p)(0,1)             ×Qjl(p,p)(1,0))]|u=u2(p−1)dv +clR12∫v01[d21Pik(p,p)(1,1)Qjl(p,p)(0,0)      +d22u2Pik(p,p)(0,0)Qjl(p,p)(1,1)      +d23u(Pik(p,p)(1,0)Qjl(p,p)(0,1)         ×Pik(p,p)(0,1)Qjl(p,p)(1,0))]|u=cldvKijkl(12)=−clR12∫v01[d21Pik(1,2)(1,1)Qjl(1,2)(0,0)        +d22u2Pik(1,2)(0,0)Qjl(1,2)(1,1)        +d23u(Pik(1,2)(1,0)Qjl(1,2)(0,1)Pik(1,2)(0,1)           ×Qjl(1,2)(1,0))]|u=clMijkl(p)=∫up−1up∫v01uPik(p,p)(0,0)Qjl(p,p)(0,0)du dv.
